# Effect of chloroquine on gene expression of *Plasmodium yoelii nigeriensis *during its sporogonic development in the mosquito vector

**DOI:** 10.1186/1475-2875-6-84

**Published:** 2007-07-02

**Authors:** Henrique Silveira, Susana Ramos, Patrícia Abrantes, Luís Filipe Lopes, Virgílio E do Rosario, Mitchell S Abrahamsen

**Affiliations:** 1 Centro de Malária e Outras Doenças Tropicais, UEI Malária, Instituto de Higiene e Medicina Tropical, Universidade Nova de Lisboa, Portugal; 2 Veterinary and Biomedical Sciences, University of Minnesota 55 108, USA

## Abstract

**Background:**

The anti-malarial chloroquine can modulate the outcome of infection during the *Plasmodium *sporogonic development, interfering with *Plasmodium *gene expression and subsequently, with transmission. The present study sets to identify *Plasmodium *genes that might be regulated by chloroquine in the mosquito vector.

**Methods:**

Differential display RT-PCR (DDRT-PCR) was used to identify genes expressed during the sporogonic cycle that are regulated by exposure to chloroquine. *Anopheles stephensi *mosquitoes were fed on *Plasmodium yoelii nigeriensis*-infected mice. Three days post-infection, mosquitoes were fed a non-infectious blood meal from mice treated orally with 50 mg/kg chloroquine. Two differentially expressed *Plasmodium *transcripts (Pyn_chl091 and Pyn_chl055) were further characterized by DNA sequencing and real-time PCR analysis.

**Results:**

Both transcripts were represented in *Plasmodium *EST databases, but displayed no homology with any known genes. Pyn_chl091 was upregulated by day 18 post infection when the mosquito had a second blood meal. However, when the effect of chloroquine on that transcript was investigated during the erythrocytic cycle, no significant differences were observed. Although slightly upregulated by chloroquine exposure the expression of Pyn_chl055 was more affected by development, increasing towards the end of the sporogonic cycle. Transcript abundance of Pyn_chl055 was reduced when erythrocytic stages were treated with chloroquine.

**Conclusion:**

Chloroquine increased parasite load in mosquito salivary glands and interferes with the expression of at least two *Plasmodium *genes. The transcripts identified contain putative signal peptides and transmembrane domains suggesting that these proteins, due to their location, are targets of chloroquine (not as an antimalarial) probably through cell trafficking and recycling.

## Background

Malaria transmission starts when the anopheline mosquito takes an infected blood meal, initiating the sporogonic cycle of the *Plasmodium *parasite. The full sporogonic cycle varies in length, depending on species and temperature, and can take up to two weeks [1]. It is speculated that since mosquitoes will have several blood meals during their life span, components of this meal can interfere with parasite development in the mosquito vector. In endemic areas anti-malarial drugs, which are taken at regular basis can end up in mosquito blood meals and, therefore, affect parasite development [2].

The anti-malarial effect of chloroquine is observed on the erythrocytic cycle of the parasite and has been associated with haem detoxification. Although chloroquine is not effective against the sporogonic cycle stages and although the parasite environment during the sporogonic cycle is not rich in haemoglobin, several reports have described that this drug can modulate parasite development within the mosquito. Early studies by Ramkaran and Peters [3] associated chloroquine treatment with increased infectivity of drug-resistant parasites to the mosquito. Later, Hogh et al. [2] reported that mosquitoes fed with *Plasmodium falciparum *or *Plasmodium berghei *gametocytes in the presence of serum from chloroquine-treated human volunteers showed four to five fold increase in the mean oocyst number and the same effect was also observed on *Plasmodium yoelii nigeriensis *N67, which displayed enhanced infectivity to mosquitoes when mice were treated with chloroquine 12 hours prior feeding [4]. Higher parasite loads were observed in salivary glands from mosquitoes that had a second blood meal containing chloroquine than those observed in non-chloroquine treated mosquitoes. This substantiates that chloroquine can modulate *Plasmodium *mosquito infection, possibly acting on both the parasite and the mosquito vector. Abrantes et al. [5] demonstrated that chloroquine can modulate the mosquito immune response, probably contributing to the infectivity differences observed. The present study shows that chloroquine can interfere with parasite development and can regulate the expression of parasite genes.

## Methods

### Experimental infections in mice

*P. y. nigeriensis *N67 were originally provided by Professor D. Walliker (Edinburgh University). All infections were performed in female BALB/C mice (four to six weeks old) (Harlan Interfauna Ibérica SL, Barcelona, Spain). Parasitized erythrocytes (PE) were derived from parasite donor mice infected by intraperitoneal (i.p.) injection of cryopreserved stabilates. Mice were infected by i.p. injection of 1 × 10^6 ^*P. y. nigeriensis *PE/mouse. Daily individual parasitaemia were determined by counting the number of parasitized erythrocytes per 5,000 red blood cells on Giemsa-stained tail-blood films. Exflagellation was investigated before mosquito feeding.

### Mosquito infections

*Anopheles stephensi *females (four to five days old) were infected by feeding on *P. y. nigeriensis*-infected mice (with patent exflagellation and 10–20% PE). Mosquitoes were allowed feeding for one to two hours. Unfed mosquitoes were discarded. Three days after infection, mosquitoes were fed a second non-infective blood meal either on BALB/c mice that were orally administrated 50 mg/Kg of chloroquine 18 hours before the mosquito blood meal, or on non-treated mice. Once again, mosquitoes were allowed feeding for one to two hours and unfed mosquitoes were discarded. Mosquitoes were provided with 10% glucose solution and 0.05% PABA, and were maintained in experimental insectaries at 24 ± 0.5°C and 70 ± 5% relative humidity. The percentage of infected mosquitoes and the number of oocyst per midgut were calculated by optical microscopy observation of midguts at day 8 p.i

### Isolation of total RNA and genomic DNA

Total RNA was isolated from mosquito midguts collected at day 8 and 18 post infection (p.i.) and from mosquito salivary glands at day 18 p.i. Tissues were kept on ice and homogenized using a mortar and pastel. After grinding TRIzol reagent (Invitrogen Corp., San Diego, Califomia) was added and total RNA was purified as described by the manufacturer. The integrity of RNA was confirmed by electrophoresis on a denaturing formaldehyde/agarose gel. Genomic DNA (gDNA) was isolated from blood stage parasites (collected from infected mice to avoid mosquito contamination) and from uninfected mosquitoes using standard phenol/chloroform and precipitated with ethanol.

### Differential mRNA display

RNA samples (1 μg total RNA) were treated with DNaseI (Invitrogen) according to the manufacturer's protocol, in order to eliminate possible DNA contamination. Complementary DNA was synthesized using a modification of the RNAimage Kit protocol (GenHunter Corp., Nashville, Tennessee). The reverse transcription reaction contained 0.4 μg of DnaseI-treated RNA, 0.2 μM anchor primer, 1× first strand buffer (50 mM Tris-HCl pH 8.3, 75 mM KCl, 3 mM MgCl_2_), 0.5 mM dithiothreitol and 20 μM dNTP's). Samples were heated to 42°C for 2 min and 200 U Superscript II reverse transcriptase (Invitrogen) was added. The samples were further incubated at 42°C for 50 min, followed by incubation at 70°C for 15 min to inactivate the reverse transcriptase.

Differential mRNA display reactions were performed as described in the RNAimage Kit protocol with the exception of reaction volume that was reduced to 10 μl. PCR mix contained 1× PCR buffer (10 mM Tris-HCl pH 8.3, 50 mM KCl; 1.5 mM MgCl_2_, 0.001% gelatin), 2 μM dNTPs, 2 μM arbitrary 5'-upstream primer, 0.2 μM anchor primer, 0.1 μl α[^33^P] dATP (1,000 Ci/mmol), 0.5 U Amplitaq polymerase (Perkin-Elmer, Foster City, Califomia), and 1 μl of first- strand cDNA. The cDNA sequences were amplified by PCR in a Perkin-Elmer model 2400 thermocycler using the following profile: 94°C for 30 sec, 40°C for 2 min, 72°C for 30 sec. Following completion of 40 cycles, the reactions were incubated at 72°C for an additional 5 min. Bands specific for *P. y. nigeriensis *that were affected by chloroquine were identified using the anchor primer H-T_11_A (5'-AAGCTTTTTTTTTTTA-3') and the 5' upstream primers H-AP18 (5'-AAGCTTAGAGGCA-3') and H-AP19 (5'-AAGCTTATCGCTC-3') obtained from GenHunter Corp. The bands were excised from the gel and reamplified by PCR using identical PCR conditions described above with the exception that α[^33^P] dATP was omitted. The reamplified cDNA fragment was purified from a 1.5% agarose gel and subcloned into the pCR2.1 vector using the TA Cloning Kit (Invitrogen).

### Dot blot analysis

Genomic dot blots were prepared using 2 μg *P. y. nigeriensis *DNA and 5 μg of *An. stephensi *DNA. The DNA was denatured by heating at 95°C for 10 min and cooled on ice. DNA was then blotted directly onto positively charged nylon membrane (Roche, Portugal) and cross-linked by UV exposure. The blots were incubated overnight at 55°C in hybridization solution (DIG Easy Hyb Granules, Roche) containing a probe labeled with DIG-dUTP by PCR (PCR DIG Probe Synthesis Kit, Roche). Filters were washed twice in 2× SSC, 0.1% (w/v) sodium dodecyl sulfate (SDS) for 5 min at room temperature and then twice in 0.1 × SSC, 0.1% SDS for 15 min at 65°C. Washing, blocking and detection were performed with the DIG Wash and Block Buffer Set (Roche), according to manufacturer instructions. After luminescent reaction with CSPD, filters were exposed to Lumi-Film Chemiluminescent Detection Film (Roche) at room temperature.

### Sequencing

Clones of interest were sequenced using the AGAC facilities at the University of Minnesota or at the STABvida cooperation (Lisbon, Portugal) using M13 universal primers. Sequences obtained were used to design gene specific primers that were used in the expression analyses by Real-Time PCR. Sequences were deposited at GeneBank under the accession numbers [GenBank: EF451823, GenBank: EF451824].

### Real-time reverse transcriptase-PCR analysis

One μg of total RNA purified from infected midguts collected at day 8 p.i and at day 18 p.i. and from infected salivary glands collected at day 18 p.i. was treated with DNase I as described above. Treated RNA was added to 13.5 μl of a solution containing 50 mM Tris-HCl pH 8.3, 75 mM KCl, 3 mM MgCl_2_, 10 mM BSA, 0.5 mM dNTP's (Promega, Southampton, UK), 0.5 μg oligodT15 (Promega, Southampton, UK), and 10 U MMLV-RT(Invitrogen). RNA was reverse transcribed 1 hr at 37 C, denatured 5 min at 95°C and cooled on ice.

PCR reactions was performed using qPCR™ Core Kit for Sybr™ Green I (Eurogentec, Seraing, Belgium) containing 1× PCR buffer, 0.2 mM dNTPs, 3.5 mM MgCl_2_, 1:66000 Sybr Green dilution, 0.5 U of Hot GoldStar enzyme and 1 μl of first-strand cDNA reaction in a total volume of 20 μl. Gene-specific primers for the *P. yoelli ldh *(5'-CCAGGAAAAAGTGACAAAGAATG-3'; forward primer 0.3 mM, 5'-AAACACCACCTAATCCAACAATC-3'; reverse primer, 0.3 mM), Pyn_chl055 (5'-AGCAGAAAAAGTTGAGGCAGTAGA-3'; forward primer 0.2 mM, 5'-GGATGTTTCAGTATATTCGGTTTTT-3'; reverse primer 0.3 mM) and Pyn_chl091 (5'-TTATGCATTTTATTACTTTTGGTGAG-3'; forward primer 0.3 mM, 5'-ATCTCTAACTTTTCATTTTCCTTGTA-3'; reverse primer, 0.3 mM) were used in the PCR reaction. Amplification and detection of specific products was performed with the GeneAmp^® ^5700 system (PE Applied Biosystems, Porto, Portugal) using the following cycle profile: one cycle at 48°C for 30 min, one cycle at 95°C for 10 min, 40 cycles at 95°C for 15 s, and 60°C for 1 min. Quantification relied on the comparison of the critical threshold cycle (Ct) of an unknown sample against a standard curve of known quantities. Ct is the amplification cycle at which the fluorescence becomes detectable and is inversely proportional to the logarithm of the initial amount of template DNA. For each reaction a standard curve was plotted with Ct values obtained from amplification of known quantities of gDNA (100, 10, 1, 0.1 and 0.01 ng) isolated from *P. y. nigeriensis *or *An. stephensi*. Concentration of DNA was determined by spectrophotometric analysis of optical density at 260 nm (GeneQuant, Pharmacia) and standard DNA solutions were prepared. The standard curves were used to transform Ct values to the relative number of DNA molecules. Triplicates of each sample and standard curve were performed in all assays. The quantity of cDNA for each experimental gene Pyn_chl055 and Pyn_chl091 was normalized to the quantity of the housekeeping gene *ldh *cDNA in each sample. Melting curves were used to determine the specificity of PCR products.

### Parasite load

To estimate the parasite burden in midguts and salivary glands of *An. stephensi*, the values obtained for the *Pyldh *gene were used after normalization to the amount obtained for the S7 gene of *A. stephensi*. To validate the quantification method, known amounts of sporozoites were used to assess the ability of the real-time PCR assay to distinguish different numbers of *P. y. nigeriensis *parasites. *An. stephensi *infected with *P. y. nigeriensis *were dissected at day 18 p.i., salivary glands were collected and homogenized in a glass tissue grinder. Sporozoites were washed and resuspended in a known volume of RPMI. The number of sporozoites was determined using a Neubauer chamber. The initial amount of 10,000 parasites was diluted ten-fold up to one parasite. The values obtained from the sporozoite titration were then correlated with the values obtained for the standard curve used during the experiment and with the Ct values obtained.

### Erythrocytic cycle

The effect of chloroquine on the expression of the genes isolated in the sporogonic cycle was analysed during the erythrocytic cycle of *P. y. nigeriensis*. C57BL/6 mice, 6–8 weeks old, were inoculated intraperitoneally with 10^7 ^PE. When parasitaemia reached 10–15%, the mice were treated with 0, 0.5, 5 and 50 mg/Kg body weight. Blood was collected at 2, 24, 48 and 72 h and was processed for RNA extraction. Blood smears were performed for each sample, in order to calculate parasitaemia. Real-time PCR analyses were performed as described for sporogonic forms.

### Statistical analysis

Statistical significance was assessed by Mann-Whitney *U*-test when comparing chloroquine-treated and non-treated-controls. Comparison between different time points was performed with the Kruskal-Wallis test, followed by multiple-comparison test. *P *values less than 0.05 were considered significant. Mann-Whitney *U *test and Kruskal-Wallis test were performed using SPSS v 13.0 for Windows (SPSS, Inc.).

## Results

**Figure 1:**
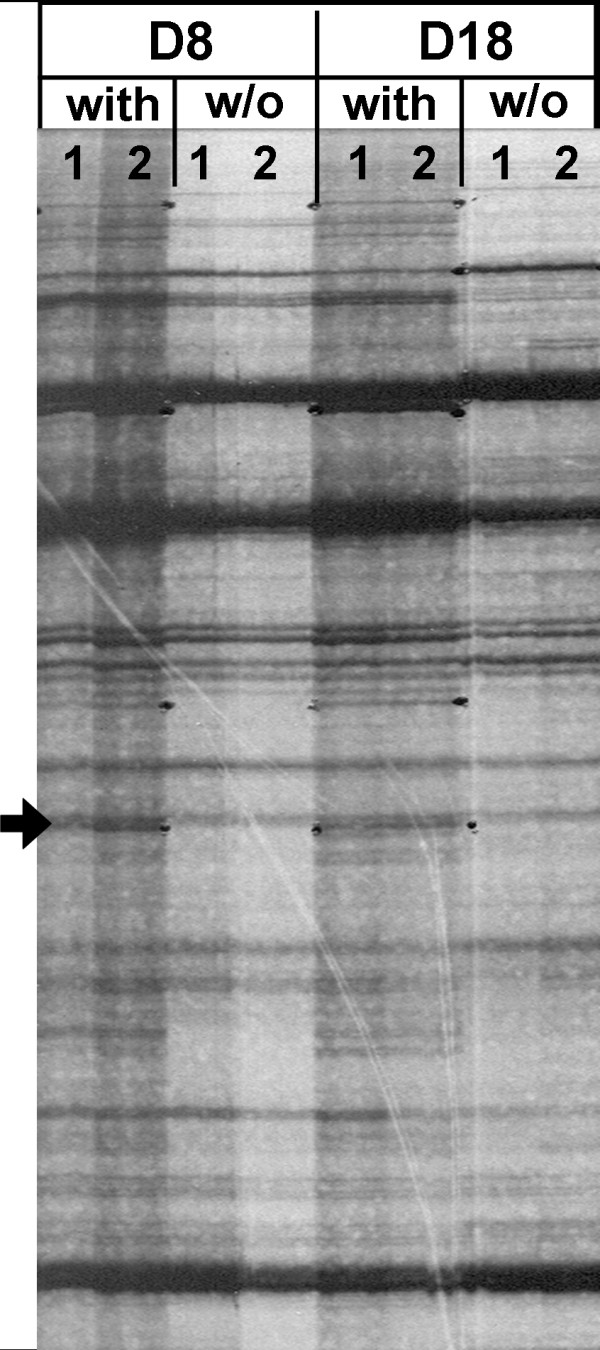
**Differential display RT-PCR analysis of total RNA isolated from infected *Anopheles stephensi *midguts**. Example of a sequencing gel. Mosquitoes had a second blood meal with or without (w/o) chloroquine at day 3 post-infection. Midguts were collected at day 8 (D8) and at day 18 (D18) post-infection. Arrow indicates the band that originated clone Pyn_chl055. Lanes 1 and 2 represent independent experiments.

The effect of a second blood meal containing chloroquine on the gene expression of *P. y. nigeriensis *during the sporogonic cycle was investigated using differential display RT-PCR (DDRT-PCR). RNA isolated from infected midguts and salivary glands was reverse transcribed and amplified by PCR using 8 mer arbitrary upstream primers. PCR products were run on a sequencing gel (Figure 1). The differentially-displayed bands were excised, re-amplified and cloned. The clones were used to generate probes for dot blot analyses, in order to determine if cDNA was of parasite or mosquito origin. This technology, allowed the isolation of a large number of differentially displayed genes that were mainly from mosquito origin. Two transcripts that hybridized solely to *P. y. nigeriensis *DNA were identified and further characterized.

**Figure 2:**
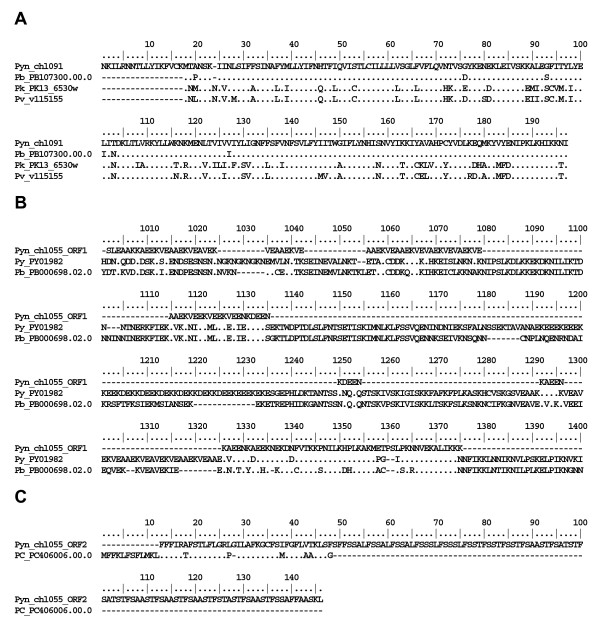
**Sequence alignments of translated sequences of Pyn_chl091 and Pyn_chl055**. Pyn_chl091 [GenBank: EF451823] (A) and Pyn_chl055 [GenBank: EF451824] (B and C) with other *Plasmodium *predicted protein sequences. PB107300.00.0, *Plasmodium berghei *hypothetical protein, gi68010828; PK13_6530w, *Plasmodium knowlesi *hypothetical protein at geneDB; v115155, *Plasmodium vivax *hypothetical protein at PlasmoDB; PY01982, mature-parasite-infected-erythrocyte-surface-antigen-putative-prot gi83282560; PB000698.02.0, hypothetical protein, gi68075557; *Plasmodium chabaudi *hypothetical protein PC406006.00.0, gi56518416. B, ORF1 [5'3'frame 2]; C, ORF2 [3'5' frame3].

The clones corresponding to differentially regulated *P. y. nigeriensis *genes (thereafter named Pyn_chl091 and Pyn_chl055) were sequenced. When the obtained sequences were BLAST compared with *P. yoelii *genome sequences at TIGR [6] an almost complete match with tentative consensus sequences was obtained (Table 1), confirming the origin of the gene obtained by dot blot analyses. Sequence similarity search was performed against TIGR, PlasmoDB and GeneDB databases, showing strong similarities with other *Plasmodium *species predicted proteins, *P. berghei*, *Plasmodium vivax*, *Plasmodium chabaudi *and *Plasmodium knowlesi *(Figure 2).

**Table 1:** BLASTn search of Pyn_chl091 and Pyn_chl055 at TIGR *Plasmodium yoelii *Gene Index* [6]

Clone	Identifier	Species**	Score	Smallest Sum Probability		Identities
				P(N)	N	
Pyn_chl091	TC8060	Pyy	2919	3.0e-151	2	601/640 (93%)
	BF295928	Pb	2457	2.1e-106	1	549/627 (87%)
	TC3830	Pv	1013	1.2e-41	1	349/545 (64%)
	BF296810	Pb	545	4.7e-20	1	113/118 (95%)
Pyn_chl055	TC7886	Pyy	489	1.2e-31	3	101/105 (96%)

* Assessed November 2006

** Pyy, *Plasmodium yoelii yoelii*; Pb, *Plasmodium berghei*; Pv, *Plasmodium vivax*

**Figure 3:**
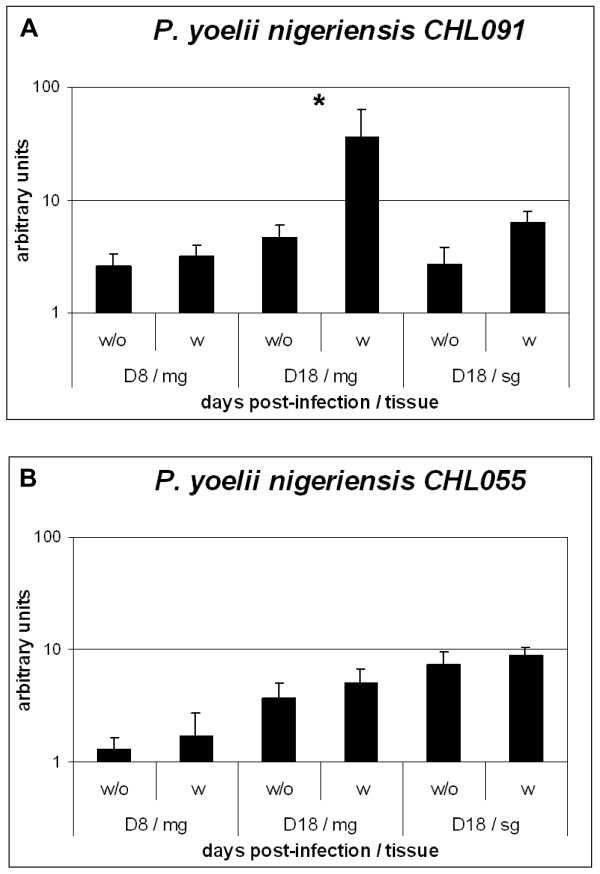
**Expression levels of two *P. y. nigeriensis *transcripts isolated by DDRT-PCR**. A) expression profile of the differential display cDNA transcripts corresponding to clone Pyn_chl091; B) expression profile of the differential display cDNA transcripts corresponding to clone Pyn_chl055. Quantification of cDNA was determined against a standard curve and normalized with the expression of *Pyldh*. Values represent the mean and standard error of 4 independent experiments. w - with chloroquine treatment; w/o - chloroquine treatment; D8 - day 8 post-infection; D18 - day 18 post-infection; mg - midgut; sg - salivary glands. * Represents statistic significance.

The obtained sequences were used to design specific primers that were used in Real-Time PCR analyses. Parasites infecting mosquitoes that had a second blood meal with chloroquine when compared with infected mosquitoes that had a second blood meal without chloroquine showed an increase expression of Pyn_chl091 (Figure 3A). Expression of Pyn_chl091 was one log higher at day 18 post-infection in mosquito midgut that had a second blood meal containing chloroquine (p = 0.039). No differences were observed between treated and non-treated at day 8 (1.2 fold increase). Parasites in salivary glands at day 18 display a 2.3 fold increase of Pyn_chl091 (p = 0.063) that was not statistically significant.

Expression of Pyn_chl055 was not markedly affected by the presence of chloroquine in the mosquito blood meal. Only a slight increase in expression was observed at day 8 and at day 18 p.i. in both midguts and salivary glands (Figure 3B). Differences in expression were observed comparing D8 with D18 midgut or salivary glands on both treated (p = 0.037, p = 0.019) and non-treated (p = 0.001 and p = 0.003). Expression of Pyn_chl055 increased five times between parasites at day eight in midguts and parasites at day 18 in salivary glands regardless of treatment.

### Parasite load

**Figure 4:**
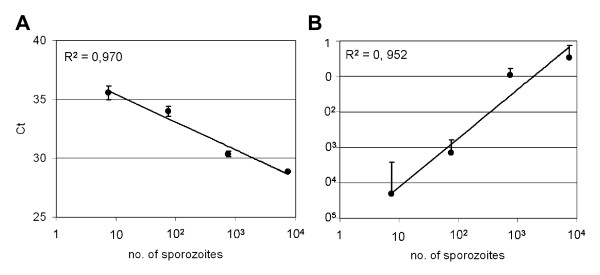
**Validation of the parasite quantification assay**. (A) Correlation between the sporozoite titration and the threshold cycles (Ct) and (B) Correlation between sporozoite titration and the values obtained with the DNA standard curve used during the Real Time PCR assays.

In order to confirm the effect of a second blood meal containing chloroquine on parasite infectivity during sporogonic cycle, parasites were quantified in two different mosquito tissues (midgut and salivary gland) using quantitative RT-PCR. Experiments were designed to assess the usefulness of this technique to detect different numbers of *P. y. nigeriensis *parasites in the mosquito vector. *P. y. nigeriensis *sporozoites were collected from infected *An. stephensi *mosquito salivary glands. The number of sporozoites was counted and serial diluted. RNA was extracted and reverse transcribed into cDNA. Real-time PCR was performed using *Pyldh *primers. At the same time the standard curve was also amplified. Correlation analyses using three experiments showed a strong correlation between the number of sporozoites and the Cts obtained and between the number of sporozoites and the standard curve used through out the experiment (Figure 4).

**Figure 5:**
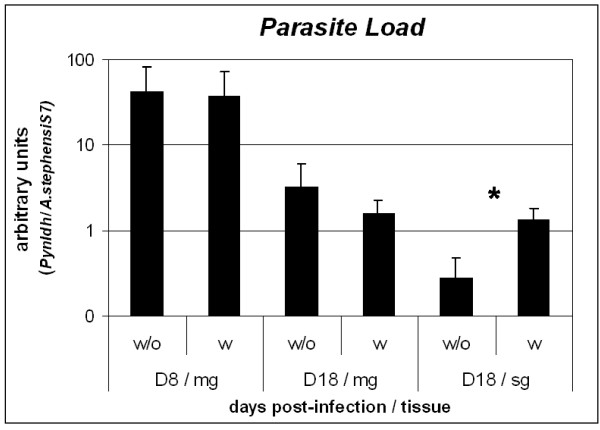
**Effect of chloroquine on the parasite load of *Anopheles stephensi *mosquitoes infected with *P. y. nigeriensis***. D8 – day 8 post-infection; D18 – day 18 post-infection; mg – midgut; sg – salivary glands. Mosquitoes had a second blood meal three days post-infection with or without chloroquine. Values represent the mean and standard error of three independent experiments. * Represents statistic significance.

To obtain parasite load in the mosquito tissue data was normalized with the expression of the mosquito constitutive gene that codes for the S7 ribosomal protein. At day 8 post infection, the number of parasites in the midgut of chloroquine-treated mosquitoes was similar to non-treated mosquitoes, but at day 18 the parasite load increased in the salivary glands (Figure 5). The highest difference between treated and non-treated mosquitoes was observed in the salivary glands at day 18, where approximately a log higher parasite load was observed (p = 0.049).

### Erythrocytic cycle

**Figure 6:**
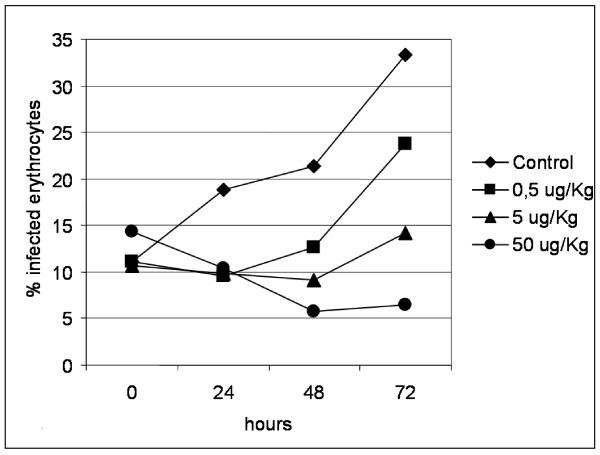
**Parasitaemia of C57Bl6 mice infected with *Plasmodium yoelii nigeriensis***. Mice were treated with 0.5, 5 and 50 mg chloroquine/Kg body weight. Values represent geometric mean of three independent experiments at 0, 24, 48 and 72 hours post-infection.

**Figure 7:**
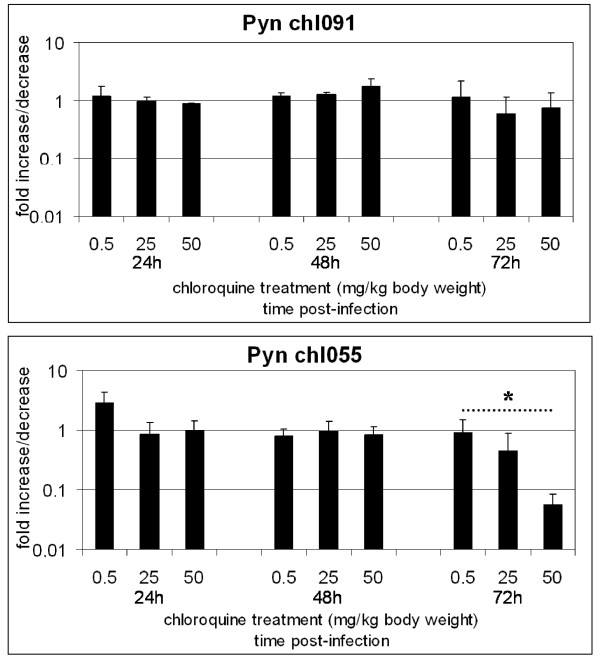
**Expression profiles of Pyn_chl091 and Pyn_chl055 during the erythrocytic cycle of *Plasmodium yoelii nigeriensis***. Quantification of cDNA was determined against a standard curve and normalized by the amount of *Pyldh *expression. Mice infected with *P. y. nigeriensis *were given orally 0.5, 25, 50 mg chloroquine/kg body weight. Blood was collected at 2, 24, 48 and 72 hours post treatment. Values represent the mean and standard error of three independent experiments. * Represents statistic significance.

In order to evaluate the effect of chloroquine treatment on Pyn_chl091 and Pyn_chl055 expression during the erythrocytic cycle, experimentally infected mice were treated with different doses of chloroquine and the expression levels were monitored at 2, 24, 48, and 72 hours. Parasitaemia decreased with treatment until 48 h in a dose-dependent manner. However, at 72 h post-infection, for all chloroquine doses parasitaemia raised as in non-treated mice (Figure 6). No major differences were observed on the expression of Pyn_chl091 or Pyn_chl055 till 48 h. At 72 h after treatment, a dose dependent decrease of Pyn_chl055 expression was observed (p = 0.038 for 50 mg/kg body weight treatment) (Figure 7).

## Discussion

The effect of chloroquine on *Plasmodium *infectivity to the mosquito has been addressed by several studies using different species and strains of *Plasmodium*. Conclusions varied, indicating either a significant increase of infection intensity [2-5,7,8] or no differences between chloroquine-treated and non-treated mosquitoes[9,10]. The present report shows higher parasite load in salivary glands of chloroquine-treated mosquitoes when compared to non-chloroquine treated mosquitoes, confirming that chloroquine can modulate *Plasmodium *infection in the mosquito.

The anti-malarial action of chloroquine is restricted to blood stages and several theories exist to explain chloroquine mechanisms of action on *Plasmodium *erythrocytic stages, including i) Intercalation into GC-rich DNA, ii) Inhibition of ornithine decarboxylase to block polyamine metabolism, iii) Inhibition of haem-dependent protein synthesis, iv) Increased vacuolar pH, v) Inhibition of vacuolar phospholipase, vi) Inhibition of haemoglobin proteases, vii) Inhibition of hydrogen peroxide degradation of haem, viii) Inhibition of glutathione degradation of haem in the cytosol and ix) Inhibition of malarial pigment formation (reviewed by Sullivan [11]). Some of these mechanisms may be oversimplified and most likely a combination of them is probably in action. However, the effect of chloroquine on the sporogonic cycle is probably of different nature as the drug does not kill parasites during this stage of development where environment and metabolism are different. Chloroquine has applications other than anti-malarial use, namely as an anti-inflammatory drug. In this context, chloroquine activity as a lysosomotropic agent has been largely documented. Most of the described effects of chloroquine can be attributed to alterations of intravesicular pH that will interfere with several membrane and recycling processes of the cell (e.g. [12,13]).

Chloroquine was fed three days after an infectious blood meal, at the time when parasites were already at the early oocyst stage. Here, the parasite multiplies at high rates in order to generate thousands of sporozoites. Given the published information on chloroquine mechanisms of action, it is expect that chloroquine might be altering the pH of oocyst intracellular vesicles, influencing trafficking and recycling, and, therefore, interfering with the production of sporozoites. The genesis of the sporozoite within the oocyst involves subdivision of cytoplasm by multiple clefts of plasmalemma forming large vesicular structures of ER origin that are called sporoblasts. These structures are covered with the circumsporozoite (CS) protein [14,15]. CS protein and GPI-anchor to this protein are essential for the formation of sporozoites [16,17]. Anchorage of GPI is done in the ER [18], and chloroquine through cell trafficking and recycling interference might unbalance this complex intra-oocyst maturation of sporozoites, leading to faster maturation of sporozoites and subsequent higher parasite load at day 18 in the salivary glands of mosquitoes that received chloroquine.

Chloroquine could also be acting directly on DNA, altering the expression of *Plasmodium *genes. Early work on this drug has shown that chloroquine can act as DNA-intercalating agent [19] and this mechanism was used to explain the antimalarial effect of the drug [20]. This is no longer accepted as the mechanisms behind anti-*Plasmodium *action, but can help understanding differences in gene expression not explained by alterations in the endolysosome milieu. It is also known that chloroquine enhances transgene expression in polycation-based, nonviral gene delivery systems and most recently data suggests that it interacts directly with nucleic acids in cells [21] facilitating this transgene expression. Even so, direct action on parasite DNA is probably minor as chloroquine tends to intercalate in C and G reach regions [20] and *Plasmodium *genome is highly reach in A and T, further the amount of chloroquine reaching the oocysts in the mosquito is far lower from that used to demonstrate chloroquine DNA intercalating action. Less likely, stability of mRNA could also have been impaired as suggested by the work of Jang and collaborators [22] in which chloroquine reduces the levels of IL-1 and IL-6 mRNA in mouse macrophages stimulated with LPS, at least in part, by decreasing their stability.

The two upregulated *P. yoelli nigeriensis *transcripts (Pyn_chl091 and Pyn_chl055) were similar to ESTs well represented in two *P. yoelii *libraries ([23] and *P. yoelii *EST project at TIGR), and showed high homology with *P. berghei *transcripts [24]. However, similarities with *P. falciparum *proteins were not very strong especially for Pyn_chl091.

The Pyn_chl091 sequence, although without a strong homology with assigned function proteins, was closely similar to other *Plasmodium*. These predicted proteins were annotated at PlasmoDB (assessed at November 2006), has having a signal peptide and transmembrane domains, suggesting that it is a membrane protein. When Pyn_chl091 ORF sequence was compared at Pfam database, only a reticulon motif was found, even so with a low predictive value (e-value of 0.089). The function of reticulon is unknown, but it has been associated with the endoplasmic reticulum (INTERPRO entry IPR003388). Chloroquine is known to act at lysosome, endosome and trans-Golgi compartments by increasing their pH and has been used to distinguish between these compartments and others that are independent of an acidic environment such as endoplasmic reticulum [25]. Knowing that chloroquine has a profound impact on cellular traffic the differences in the transcription profiles of Pyn_chl091, a putative membrane protein, are probably a result of this effect.

The Pyn_chl055 sequence was similar to putative protein named mature-parasite-infected erythrocyte surface antigen. Its name is not in consonance with its expression at the sporogonic cycle. Expression of Pyn_chl055 increases towards the end of the sporogonic cycle being highest at the salivary glands. Chloroquine had no major impact on the transcript abundance of Pyn_chl055 during the sporogonic cycle, but treatment of blood stages with chloroquine down-regulated the expression of this gene, suggesting that it might be regulated by chloroquine treatment during the erythrocytic cycle, further studies are needed to confirm its involvement on chloroquine anti-malarial effect.

## Conclusion

Membrane proteins can play an important role on the regulation of drug accumulation and trafficking and chloroquine induction of *Plasmodium *transcripts for encoding membrane proteins and transporters have been described in other studies [26]. *P. yoelii nigeriensis *genes, up-regulated when mosquitoes received a second blood meal on chloroquine-treated mice, were identified and associated with increased infectivity to the mosquito. Further, one of these genes was also regulated by the presence of chloroquine during erythrocytic cycle. Both transcripts seem to code for membrane associated proteins suggesting that these proteins due to their location are targets of chloroquine action probably through membrane trafficking that may increase their retention at the cell surface. Depending on their function this phenomenon could have implications on drug concentration within the cell and subsequent effect of the drug on the infected erythrocyte. This study shows the importance of some antimalarial drugs in activity non related to the blood cycle though affecting possibly and unexpectedly malaria transmission.

## Authors' contributions

HS conceived and designed the experiments; HS, SR, PA and LFL performed the experiments; HS, SR and MSA, analysed and interpreted the data; VER contributed with reagents/materials/analysis tools and critical review of the manuscript. HS and MSA wrote the paper. All authors read and approved the final manuscript.
